# Safety and Viral Shedding of Live Attenuated Influenza Vaccine (LAIV) in Chinese Healthy Juveniles and Adults: A Phase Ⅰ Randomized, Double-Blind, Placebo-Controlled Study

**DOI:** 10.3390/vaccines10111796

**Published:** 2022-10-26

**Authors:** Li Li, Nianmin Shi, Na Xu, Haibin Wang, Hui Zhao, Haidong Xu, Dawei Liu, Zheng Zhang, Shuping Li, Junnan Zhang, Chunhui Guo, Jinglei Huo, Menghan Zhao, Fengji Luo, Liqing Yang, Yunhua Bai, Qiang Lu, Yusong Zhang, Yi Zhong, Wenhui Gao

**Affiliations:** 1 Chaoyang District Center for Disease Control and Prevention, No. 25 Huaweili, Panjiayuan, Chaoyang District, Beijing 100021, China; 2 Vaccine Clinical Research Branch of China Association for Vaccines, No. 11, Xinzhong St., Dongcheng District, Beijing 100010, China; 3 Changchun BCHT Biotechnology Co., No. 138 Zhuoyue St., Gaoxin District, Changchun 130012, China; 4 National Institutes for Food and Drug Control, No. 31, Huatuo Avenue, Daxing Biomedical Complex, Daxing District, Beijing 102600, China; 5 Jinsong Community Health Service Center, No. 501, Jinsong St., Chaoyang District, Beijing 100020, China; 6 Baotou Medical College, No. 31 Jianshe Rd., Donghe District, Baotou 014000, China; 7 Xiangheyuan Community Health Service Center, No. 15 Liufang St., Chaoyang District, Beijing 100020, China; 8 Beijing Institute of Biological Products, No. 13, South East Third Ring Rd., Chaoyang District, Beijing 100021, China

**Keywords:** live attenuated influenza vaccine, safety, viral shedding

## Abstract

This study was a randomized, double-blind, placebo-controlled study to evaluate the safety and viral shedding of live attenuated influenza vaccine (LAIV) in Chinese healthy juveniles and adults. A total of 80 Eligible volunteers were divided into two age groups (≥18 and 3–17 years old). Volunteers were randomly and equally assigned to the experimental group and placebo-controlled group by ratio of 3:1 in each age group. Vaccination was carried out in steps. Totally, 34 (56.67%) adverse events and 24 (40.00%) adverse reactions of the LAIV group were reported. Most adverse reactions were grade 1 and grade 2, and the incidence of adverse reactions that grade 3 was 5%. The most common local reaction was runny nose/nasal congestion (n = 4, 6.67%). And the most common general reaction was fever (n = 10, 16.67%). There were no statistically significant differences in the incidence of total adverse reactions, different grades of adverse reactions, and symptoms between the experimental group and placebo-controlled group. No severe adverse events were reported. Three subjects (5.00%) had been detected vaccine strains on the 3rd day after LAIV vaccination; one was type B and the other two were H3N2. Four subjects (6.67%) had been detected with vaccine strains on the 7th day after LAIV vaccination, all were H3N2. There were no subjects detected carrying the influenza virus on the 15th day after vaccination. There were no statistically significant differences in the positive rate of vaccine strains of influenza virus between the experimental group and placebo-controlled group. The vaccine was well tolerated and not associated with increased rates in adverse reactions or the occurrence of severe adverse events. Pathogenicity of shed vaccine virus to surrounding people was not observed. Thus, Phase Ⅱ study can be carried out as scheduled.

## 1. Introduction

Influenza is an acute viral respiratory infection [1]. It is still one of the major public health issues because it can cause an annual epidemic and can potentially instigate a global pandemic [2]. Most persons who become ill after the influenza virus infection recover without serious complications and sequelae. However, influenza can be associated with serious illnesses, hospitalizations, and deaths, particularly among older adults, young children, pregnant women, and persons with certain chronic diseases [3]. Influenza is also an important cause of missing work and school [3]. Generally, the vaccination is considered the most effective approach to avoid or reduce infection from the seasonal influenza virus [4]. However, despite the almost worldwide vaccination plan of influenza, there are still 1 billion individuals infected with the influenza virus annually. Meanwhile, influenza causes 3–5 million cases of severe diseases, and 290,000 to 650,000 deaths every year, according to World Health Organization (WHO) and Center for Disease Control (CDC) [5].

Nowadays, there are two major types of influenza vaccine: inactivated influenza vaccine (IIV) and live attenuated influenza vaccine (LAIV) [1]. The IIV is approved for use in individuals aged more than six months, including persons with certain chronic diseases and pregnant women. It is administered by intramuscular injection [6]. LAIV is approved in the USA for use in healthy individuals 2–49 years old [7], and in Europe for individuals 2–18 years old [8]. The most significant advantage of LAIV is the non-invasive route of administration by nasal spray. Furthermore, it imitates the natural infection, stimulating mucosal and humoral immunity. Hence, LAIV is considered the most suitable candidate for mass immunisation, especially in a pandemic [9]. 

In China, “GanWu” (Influenza vaccine, Live, Nasal, Freeze-dried) is the first live attenuated influenza vaccine developed by Changchun BCHT Biotechnology Co. In April 2020, it was approved by the National Medical Products Administration of China (NMPA). “GanWu” is produced by Influenza A virus and Influenza B virus cultured in chick embryo cells. In the preclinical phase, the abnormal toxicity test, acute toxicity test, and preliminary stability test have achieved relevant regulations by NMPA. To evaluate the safety, viral shedding, immunogenicity, and efficacy of “GanWu”, phase Ⅰ, Ⅱ, and Ⅲ studies have been carried out, respectively. This paper reports the findings in the phase Ⅰ study. 

## 2. Materials and Methods

### 2.1. Study Design

This study was a randomized, double-blind, placebo-controlled study to evaluate the safety and viral shedding of LAIV in Chinese healthy juveniles and adults.

This clinical trial was performed abide by the Declaration of Helsinki and Good Clinical Practice (GCP). And before vaccination was carried out, the study protocol, recruitment materials, informed consent form, and other study-related documents were approved by the ethics committee of Chaoyang District Center for Disease Control and Prevention.

Chinese healthy juveniles and adults, over 3 years old, without a history of influenza in nearly three months and influenza vaccination in the current influenza season, were eligible in the study. The exclusion criteria included allergy to any vaccine; fever (axillary temperature was higher than 37 °C); acute disease or infection; treatment with immunosuppressive or immune enhancer drugs; congenital malformation, developmental disorder; serious chronic diseases; asthma; mental, heart, liver, kidney, blood, and other organ system diseases or functional disorders; blood or blood products transfusion (within 3 months); antituberculosis therapy; pregnancy, planned pregnancy during the study; other conditions not compliable with the study protocol. Written informed consents were obtained from all volunteers before enrollment.

**Figure 1:**
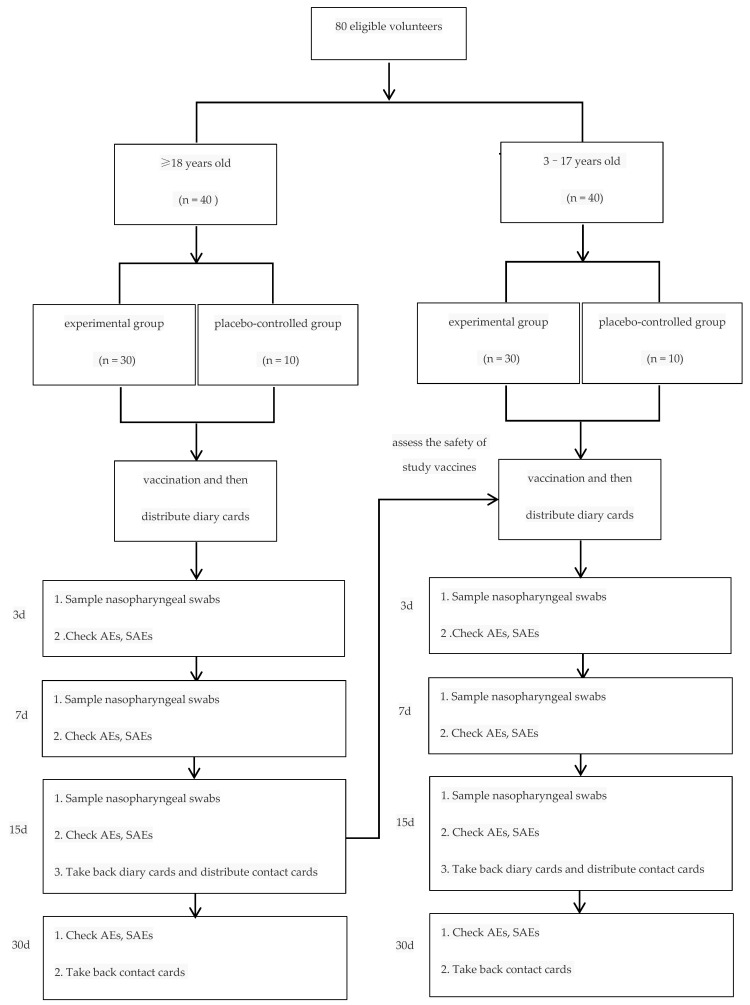
Diagram for study design. (1. Volunteers were randomly assigned to experimental and placebo-controlled groups by a ratio of 3:1; 2. 3–17 years old were permitted to be vaccinated only when the incidences of grade 3 and grade 4 adverse events lower than 15% at 14 days after vaccination were confirmed).

Eligible volunteers were divided into two age groups, ≥18 and 3–17 years old. Then volunteers were randomly and equally assigned to the experimental and placebo-controlled groups by a ratio of 3:1 in each age group. Vaccination was carried out in steps. Volunteers ≥ 18 years old were vaccinated and monitored by investigators firstly; 3–17 years old were permitted to be vaccinated only when the incidences of grade 3 and grade 4 adverse events lower than 15% at 14 days after vaccination were confirmed (Figure 1).

### 2.2. Vaccines 

The experimental vaccine (contained 7.8 and 7.9 Lg EID50 of live attenuated influenza A H1N1 and H3N2 viruses, respectively, and 7.6 Lg EID50 of type B viruses, 0.2mL/dose, Lot No. 201509T01) and the placebo-controlled vaccine (0.2mL/dose, Lot No. 201509T02) were manufactured by Changchun BCHT Biotechnology Co., Changchun, China. Both the experimental vaccine and placebo-controlled vaccine were administered by nasal spray, 0.1ml for each nasal cavity.

### 2.3. Randomization and Masking

Vaccines randomization, blinding and repackaging were performed by professional statisticians, who were unrelated to the study. The blinding code was sealed and kept by the main investigator until the database was locked. 

Each subject was allocated a unique study number in the study and the study number was the same as the number printed on the package of each vaccine. The allocation of vaccines determined the allocation of subjects. All investigators and subjects were blind to the vaccines. 

### 2.4. Safety Assessment

Immediate adverse events were monitored and recorded by the investigators at 30 min after vaccination. After that, all symptoms were recorded on diary cards and contact cards by the subjects and reviewed by the investigators until 30 days after vaccination. Adverse events (AEs) in this study referred to any adverse medical events (including events unrelated to study vaccines) during the study period. Of all AEs, those related to study vaccines were defined as adverse reactions (ARs). The association of AEs with vaccines was determined by investigators. 

Adverse events were collected and graded according to “Guidelines for grading standards for adverse reactions in clinical trials of vaccines for prophylactic use” (NMPA) and “Common Terminology Criteria for Adverse Events (V 4.0)” (United States Department of Health and Human Services, HHS). Generally, grade 1 adverse events were considered as mild, grade 2 as moderate, grade 3 is severe, and grade 4 as potentially life-threatening [10,11]. 

### 2.5. Viral Shedding

Nasopharyngeal swabs were sampled on days 3, 7 and 15 after vaccination. Influenza virus RNA was extracted from nasopharyngeal swabs by QIAamp Viral RNA Mini Kit (Qiagen, Germany) at the laboratory of Chaoyang District Center for Disease Control and Prevention. The nucleic acid typing (H1N1, H3N2, B-Victoria, and B-Yamagate) of the influenza virus was performed by multiplex PCR. Then genetic sequencing was conducted to determine whether the influenza virus was one of vaccine strains or naturally infected strains.

### 2.6. Assessment Indicators

Indicators for safety included the incidence of adverse events, adverse reactions, and severe adverse events (SAEs). Viral shedding was determined by the positive rate of vaccine strains of influenza virus on days 3, 7 and 15 after vaccination.

### 2.7. Statistical Analyses

According to “Technical Guidelines for vaccine clinical trials” (NMPA), this phase Ⅰ study enrolled 80 volunteers, including 60 in the experimental group. 

SAS software was used for statistical analysis. Measurement data was described by mean and standard deviation, and enumeration data was calculated by frequency or percentage. Fisher’s exact test was used to compare the difference of the incidence of AEs, ARs, SAEs and the positive rate of vaccine strains of influenza virus between the experimental group and placebo-controlled group. The level of significance was *p* < 0.05.

In this study, the safety set (SS) was used for analysing the safety of study vaccines, including all of the cases who had been enrolled and vaccinated. The number of one type of adverse event/reaction was calculated by the number of subjects who had this symptom.

## 3. Results

### 3.1. Study Population

After screening among 126 volunteers, 80 eligible were enrolled. They were divided into two age groups: 40 for ≥18 years old and 40 for 3–17 years old. In each age group, 30 were randomized into the experimental group and 10 into the placebo-controlled group. All subjects were included in SS.

The demographics characteristics of subjects are shown in Table 1. There were no statistically significant differences in age and gender between groups.

### 3.2. Safety

Totally, 34 (56.67%) adverse events and 24 (40.00%) adverse reactions of the LAIV group were reported. The details of adverse events and adverse reactions are shown in Table 2. Most adverse reactions were grade 1 and grade 2, and the incidence of adverse reactions that grade 3 was 5% (Table 3). The most common local reaction was runny nose/nasal congestion (n = 4, 6.67%). And the most common general reaction was fever (n = 10, 16.67%). There were no statistically significant differences in the incidence of total adverse reactions, different grades of adverse reactions and symptoms between the experimental group and placebo-controlled group.

Almost all adverse reactions occurred in 0 to 14 days after the LAIV vaccination (n = 23, 38.33%). Besides, there was only 1 adverse reaction recorded in 15 to 30 days after vaccination (Table 4). 

Throughout the observation period, no severe adverse events were reported.

### 3.3. Viral Shedding

Three subjects (5.00%) had been detected vaccine strains on the 3rd day after LAIV vaccination; one was type B and the other two were H3N2. Four subjects (6.67%) had been detected vaccine strains on the 7th day after LAIV vaccination, all were H3N2. There were no subjects had been detected carrying influenza virus on the 15th day after vaccination. There were no statistically significant differences in the positive rate of vaccine strains of influenza virus between the experimental group and placebo-controlled group (Table 5).

The details of subjects shed virus are shown in Table 6.

## 4. Discussion

WHO position paper notes that LAIV has been routinely used in Russian, USA, Canada, European Union and the United Kingdom for several years [12]. “GanWu” as the research vaccine in this study is the first approved LAIV by NMPA in China. This is the first study to evaluate the safety and viral shedding of LAIV in Chinese healthy juveniles and adults.

LAIV has many advantages. LAIV imitates natural infection, stimulating mucosal and humoral immunity without causing severe illness. LAIV was produced by growing attenuated influenza viruses in chick embryo cells [13]. These attenuated viruses are temperature sensitive. They can only grow at 25 °C (cold-adapted) which is same as the temperature of nasopharynx’s mucosal surface. Thus, attenuated viruses in LAIV can grow on the mucosal surface to stimulate mucosal immunity. However, IIV can only develop humoral immunity [14,15,16]. Besides, LAIV was administrated through nasal mucosa, which can dramatically improve the acceptability and compatibility of recipients, especially in children. Meanwhile, LAIV also has several limits. Due to the risk of using live viruses for immunization, LAIV is not recommended for immunocompromised individuals with low immunity or people who are in close contact with them [2]. Because LAIV is a live-virus vaccine and data on its administration to pregnant women and the associated maternal and fetal risks are limited, LAIV is also not recommended during pregnancy [12].

In this phase Ⅰ study, we evaluated the safety of LAIV in subjects that ≥18 and 3–17 years old, respectively. Total incidence of adverse reactions was 40.00% in the LAIV group, 36.67% for ≥18 years old group and 43.33% for 3–17 years old group. Most adverse reactions were grade 1 and grade 2, and the total incidence of adverse reactions that grade 3 was 5% in the LAIV group, 10.00% for ≥18 years old group and 0.00% for 3–17 years old group. There were no statistically significant differences in the incidence of adverse reactions and different grades of adverse reactions between the experimental group and placebo-controlled group. These results were highly consistent with findings of other studies reported [17,18,19,20,21]. 

According to WHO position paper, LAIV has generally been well tolerated in healthy children and adults. When symptoms do occur, they include self-limiting mild nasal congestion or runny nose, sore throat and low-grade fever [12]. In this phase Ⅰ study, the most common local reaction was runny nose/nasal congestion (6.67% in the LAIV group), and the most common general reaction was fever (16.67% in the LAIV group). The results of this study were the same as WHO position paper. There was one point need to be noticed: sore throat was not the major symptom in this study, the incidence of sore throat was very low (1.67% in the LAIV group). Analyzing the reasons, which may be related to the limitations of this study, will be elaborated below. 

Stan L Block’s study showed that 17–44% of subjects aged 5–49 years old shed the vaccine virus after LAIV vaccination. Shedding occurred on days 1–11 postvaccination. Shedding incidence peaked on day 2. Shedding was related to the age of the subjects and the infection status of the subject at the time of vaccination [22]. In this study, the incidence of viral shedding was 5% on the 3rd day, and 6.67% on the 7th day after LAIV vaccination. No subject was detected carrying influenza virus on the 15th day after vaccination. H3N2 was the dominant strain, followed by B. There was no H1N1 been detected. The incidence of viral shedding was 3.33% in ≥18 years old group, and 16.67% in 3–17 years old group, which suggested that viral shedding may be age-related, juveniles were more than adults. There was 1 (4.00%) female subject shedding the LAIV vaccine virus, and the ratio was 14.29% in male subjects, which suggested that viral shedding may be related to gender; men were more likely to exhibit viral shedding than women. Influenza virus reassortment and atavism were not found in the study. Pathogenicity of the shed vaccine virus to surrounding people was not observed, also. As described in WHO position paper, the transmission of the vaccine virus to nonimmune persons appears to be rare and is of no public health significance [12].

This study has several limitations. Firstly, the sample size was small, and the study period was short; therefore, the safety cannot be fully evaluated. Phase Ⅱ and Ⅲ studies will be needed for further evaluation. Secondly, in this study, nasopharyngeal swabs were sampled by a single nasal cavity of each subject. Maybe this was the probable reason why the incidence of viral shedding was lower than reported. This point will be considered in Phase Ⅱ and Ⅲ studies.

## 5. Conclusions

The vaccine was well tolerated and not associated with increased rates of adverse reactions or the occurrence of severe adverse events. Pathogenicity of shed vaccine virus to surrounding people was not observed. Thus, Phase Ⅱ study can be carried out as scheduled.

## Figures and Tables

**Table 1:** Demographics characteristics of study participants.

Characteristic	LAIV Group (n = 60/30)	Placebo-Controlled Group (n = 20/10)	Statistic/*p*
Age (mean ± SD)			
Overall	23.42 ± 17.33	21.58 ± 15.17	0.6730
≥18 years old	38.75 ± 10.54	34.37 ± 10.23	0.2593
3–17 years old	8.09 ± 3.76	8.78 ± 4.15	0.6253
Gender (n, %)			
Overall			
male	35 (58.33)	11 (55.00)	0.7940
female	25 (41.67)	9 (45.00)	
≥18 years old			
male	16 (53.33)	5 (50.00)	1.0000
female	14 (46.67)	5 (50.00)	
3–17 years old			
male	19 (63.33)	6 (60.00)	1.0000
female	11 (36.67)	4 (40.00)	

Analysis of variance (ANOCVA) was used for the comparison between groups of age, and Fisher’s exact text probability was used for the comparison between groups of gender.

**Table 2:** Adverse events and details of adverse reactions (SS).

	LAIV Group (n = 60/30)	Placebo-Controlled Group (n = 20/10)	Statistic/*p*
Total AE, n (%)			
Overall	34 (56.67)	8 (40.00)	0.3715
≥18 years old	12 (40.00)	8 (80.00)	0.6650
3–17 years old	22 (73.33)	0 (0.00)	0.0381
Total AR, n (%)			
Overall	24 (40.00)	8 (40.00)	0.7498
≥18 years old	11 (36.67)	8 (80.00)	0.3878
3–17 years old	13 (43.33)	0 (0.00)	0.1612
General reactions, n (%)	17 (28.33)	4 (20.00)	1.0000
Fever (≥37.1 °C)			
Overall	10 (16.67)	0 (0.00)	0.1906
≥18 years old	4 (13.33)	0 (0.00)	0.5558
3–17 years old	6 (20.00)	0 (0.00)	0.5558
Headache			
Overall	3 (5.00)	1 (5.00)	1.0000
≥18 years old	2 (6.67)	1 (10.00)	1.0000
3–17 years old	1 (3.33)	0 (0.00)	1.0000
Fatigue			
Overall	2 (3.33)	1 (5.00)	1.0000
≥18 years old	1 (3.33)	1 (10.00)	0.4423
3–17 years old	1 (3.33)	0 (0.00)	1.0000
Myalgia			
Overall	1 (1.67)	0 (0.00)	1.0000
≥18 years old	1 (3.33)	0 (0.00)	1.0000
3–17 years old	0 (0.00)	0 (0.00)	-
Cough			
Overall	1 (1.67)	2 (10.00)	0.1526
≥18 years old	0 (0.00)	2 (20.00)	0.0577
3–17 years old	1 (3.33)	0 (0.00)	1.0000
Local reactions, n (%)	5 (8.33)	3 (15.00)	1.0000
Runny nose/Nasal congestion			
Overall	4 (6.67)	1 (5.00)	1.0000
≥18 years old	3 (10.00)	1 (10.00)	1.0000
3–17 years old	1 (3.33)	0 (0.00)	1.0000
Sore pharynx			
Overall	1 (1.67)	1 (5.00)	0.4399
≥18 years old	0 (0.00)	1 (10.00)	0.2500
3–17 years old	1 (3.33)	0 (0.00)	1.0000
Sore larynx			
Overall	0 (0.00)	1 (5.00)	0.2500
≥18 years old	0 (0.00)	1 (10.00)	0.2500
3–17 years old	0 (0.00)	0 (0.00)	-
Infectious and infective diseases, n (%)	1 (1.67)	0 (0.00)	1.0000
Nasopharyngitis			
Overall	1 (1.67)	0 (0.00)	1.0000
≥18 years old	0 (0.00)	0 (0.00)	-
3–17 years old	1 (3.33)	0 (0.00)	1.0000
Disease of respiratory system, chest and mediastinum, n (%)	1 (1.67)	1 (5.00)	0.4399
Nasal discomfort			
Overall	0 (0.00)	1 (5.00)	0.2500
≥18 years old	0 (0.00)	1 (10.00)	0.2500
3–17 years old	0 (0.00)	0 (0.00)	-
Epistaxis			
Overall	1 (1.67)	0 (0.00)	1.0000
≥18 years old	0 (0.00)	0 (0.00)	-
3–17 years old	1 (3.33)	0 (0.00)	1.0000

The number of adverse events or reactions represents the number who had at least one adverse event or reaction of this symptom. For example, a participant with several events or reactions would be calculated once in one symptom and would also be calculated once in another symptom.

**Table 3:** Grading of adverse reactions (SS).

AR	LAIV Group (n = 60/30)	Placebo-Controlled Group (n = 20/10)	Statistic/*p*
Grade 1, n (%)			
Overall	15 (25.00)	5 (25.00)	1.0000
≥18 years old	6 (20.00)	5 (50.00)	0.3878
3–17 years old	9 (30.00)	0 (0.00)	0.3074
Grade 2, n (%)			
Overall	6 (10.00)	2 (10.00)	1.0000
≥18 years old	2 (6.67)	2 (20.00)	1.0000
3–17 years old	4 (13.33)	0 (0.00)	1.0000
Grade 3, n (%)			
Overall	3 (5.00)	1 (5.00)	0.4399
≥18 years old	3 (10.00)	1 (10.00)	0.4423
3–17 years old	0 (0.00)	0 (0.00)	-

**Table 4:** Analysis of the time of occurrence of adverse reactions (SS).

AR	LAIV Group (n = 60/30)	Placebo-Controlled Group (n = 20/10)	Statistic/*p*
Within 30 min, n (%)			
Overall	0 (0.00)	1 (5.00)	0.2500
≥18 years old	0 (0.00)	1 (10.00)	0.2500
3–17 years old	0 (0.00)	0 (0.00)	-
0–14 day, n (%)			
Overall	23 (38.33)	8 (40.00)	1.0000
≥18 years old	11 (36.67)	8 (80.00)	0.3878
3–17 years old	12 (40.00)	0 (0.00)	0.3074
15–30 day, n (%)			
Overall	1 (1.67)	0 (0.00)	1.0000
≥18 years old	0 (0.00)	0 (0.00)	-
3–17 years old	1 (3.33)	0 (0.00)	1.0000

The number of adverse reactions that occurred at 0–14 days after vaccination includes the number of adverse reactions that occurred within 30 min.

**Table 5:** Viral shedding of subjects.

Vaccine Strains	LAIV Group (n = 60/30)	Placebo-Controlled Group (n = 20/10)	Statistic/*p*
3 day, n (%)			
Overall	3 (5.00)	0 (0.00)	1.0000
≥18 years old	1 (3.33)	0 (0.00)	1.0000
3–17 years old	2 (6.67)	0 (0.00)	1.0000
7 day, n (%)			
Overall	4 (6.67)	0 (0.00)	1.0000
≥18 years old	0 (0.00)	0 (0.00)	-
3–17 years old	4 (13.33)	0 (0.00)	1.0000
15 day, n (%)			
Overall	0 (0.00)	0 (0.00)	-
≥18 years old	0 (0.00)	0 (0.00)	-
3–17 years old	0 (0.00)	0 (0.00)	-

**Table 6:** Details of subjects shed virus.

Subjects	Age (y)	Gender	Viral Shedding		
			3 Day	7 Day	15 Day
1	39.4	female	B	-	-
2	4.6	male	H3N2	H3N2	-
3	6.5	male	H3N2	-	-
4	4.6	male	-	H3N2	-
5	4.7	male	-	H3N2	-
6	3.5	male	-	H3N2	-

## Data Availability

Not applicable.

## Abbreviations

LAIV	live attenuated influenza vaccine
WHO	World Health Organization
CDC	Center for Disease Control
NMPA	National Medical Products Administration of China
GCP	Good Clinical Practice
HHS	United States Department of Health and Human Services
AEs	adverse events
ARs	adverse reactions
SAEs	severe adverse events
SS	safety set
